# Recurrent left atrial myxoma in Carney complex: A case report of a familial pedigree

**DOI:** 10.1097/MD.0000000000010247

**Published:** 2018-03-23

**Authors:** Liaoyuan Wang, Qing Wang, Yue Zhou, Qian Xue, Xiao Sun, Zhinong Wang, Guangyu Ji

**Affiliations:** Department of Cardiothoracic Surgery, Changzheng Hospital, the Second Military Medical University, Shanghai, China.

**Keywords:** cardiac myxoma, Carney complex, PRKAR1A gene

## Abstract

**Rationale:**

Carney complex (CNC) accounts for up to two-thirds of familial cardiac myxoma, which is a rare autosomal dominant syndrome characterized by multiple mucocutaneous lesions and endocrine tumors. Mutation in the cAMP-dependent protein kinase A (PKA) regulatory (R) subunit 1 (PRKAR1A) gene has been identified as a cause of CNC. In this article, we report 3 first-degree relatives with cardiac myxoma who were diagnosed with CNC and underwent surgical resection.

**Presenting concerns:**

The recurrence of cardiac myxoma was detected in a 45-year-old male by echocardiography 5 years after the resection was carried out, without any additional symptoms. Family screening indicated that his brother and his brother's son also had a history of cardiac myxoma.

**Diagnosis:**

The echocardiography of the patient showed a 43 mm × 28 mm echo mass at the bottom of the atrial septum near anterior mitral leaflet. Sequencing of the patient's genomic DNA obtained from peripheral blood identified a p.E17X (c.491-492delTG) mutation in PRKAR1A, which encodes the type Iα regulatory subunit of protein kinase A.

**Interventions:**

The patient received redo cardiac myxoma resection and mitral valve repair under cardiopulmonary bypass. Echocardiographic surveillance was conducted after the surgery.

**Outcomes:**

The patient recovered quickly after the surgery and was discharged without any abnormality detected by echocardiography. Follow-up after 1 year showed no recurrence of the cardiac myxoma.

**Main lesson:**

We recommend echocardiographic surveillance of the affected individuals and their first-degree relatives at regular intervals, given the high risk of recurrence and the morbidity and mortality associated with cardiac tumors in any location.

## 1 Introduction

Carney complex (CNC) is a rare syndrome characterized by pigmented skin lesions, multiple endocrine, cardiac myxoma, and other tumors.^[1]^ Mostly examined as a results of an incidental finding, patients with cardiac myxoma may still present with obstructive and/or embolic phenomena associated with nonspecific constitutional symptoms, including fatigue, fever, and arthralgia.^[2]^ The treatment is surgical excision, and upon detection, should be performed expeditiously given the risk of sudden death or serious embolic complications.^[3]^

However, there is little knowledge about CNC in Asian populations, especially a familial report. In this article, we report a patient (proband) who presented with left atrial myxoma. He underwent surgical resections in our cardiac surgical unit, twice across a 5-year period. Up on investigation, 2 members of his family were found to have the same disease. Hence, this article serves to illustrate the pertinent features of the diagnosis and management of familial atrial myxoma associated with CNC, and to highlight the importance of interval surveillance.

## 2 Case report

### 2.1 Institutional review board statement

The study was reviewed and approved by the ethics committee of the Changzheng Hospital, Second Military Medical University. Informed consent was obtained from the patient and his family.

### 2.2 Patient features

A 45-year-old male was diagnosed with recurrent cardiac myxoma in a local hospital and was sent to our center for further treatment. Five years ago, the patient was admitted into the Changhai Hospital with the main complaint of exertional palpitation and was diagnosed with left atrial cardiac myxoma. He then received the routine myxoma resection under cardiopulmonary bypass.

**Figure 1:**
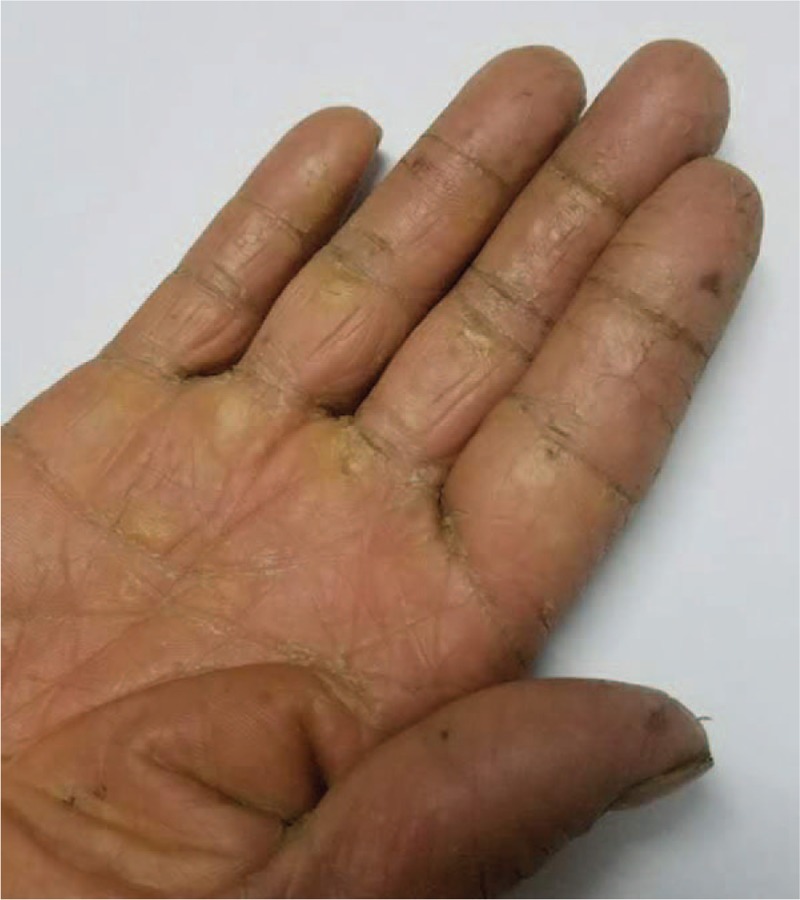
Pigmentation on the right hand of the patient.

On physical examination, extensive pigmentation was observed on his upper lip and right hand (Fig. 1). Upon palpation, no nodules were observed in the thyroid. No pathological murmurs of the heart were observed through auscultation.

**Figure 2:**
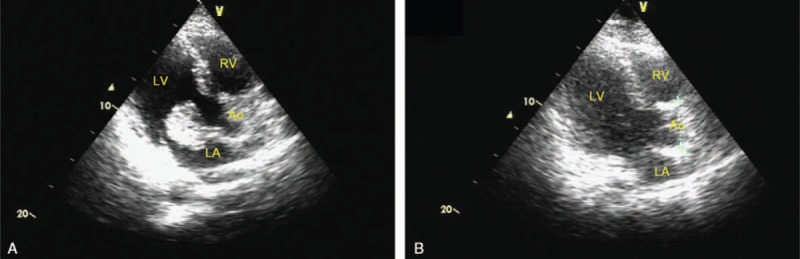
Left ventricular long axis view of TTE before and after the surgery. (A) A 43 mm × 28 mm echo mass attached on the anterior mitral leaflet; (B) the tumor was resected. TTE = transthoracic echocardiography.

A thyroid ultrasonography detected multiple small hypoechoic thyroid nodules, and the patient was euthyroid. Transthoracic echocardiography (TTE) showed a 43 mm × 28 mm echo mass in the left atrium, with the pedicle attached to the bottom of the atrial septum near the anterior leaflet, which moved with the cardiac cycle (Fig. 2A).

### 2.3 Treatment and outcomes

Routine examinations were conducted before the surgery to screen other complications. After intubation and anesthesia, conventional median sternotomy was chosen as the incision, and cardiopulmonary bypass was established. The right atrium was entered and the tumor was removed, with mitral valve annuloplasty. After the surgery, the patient was transferred to the CICU. He recovered well and was discharged soon. Postoperative TTE showed that the tumor was removed and the mitral valve functioned well (Fig. 2B). Six-month and 1-year follow-up of the patient showed no recurrence of the cardiac myxoma by TTE.

### 2.4 Other patients in this family

**Figure 3:**
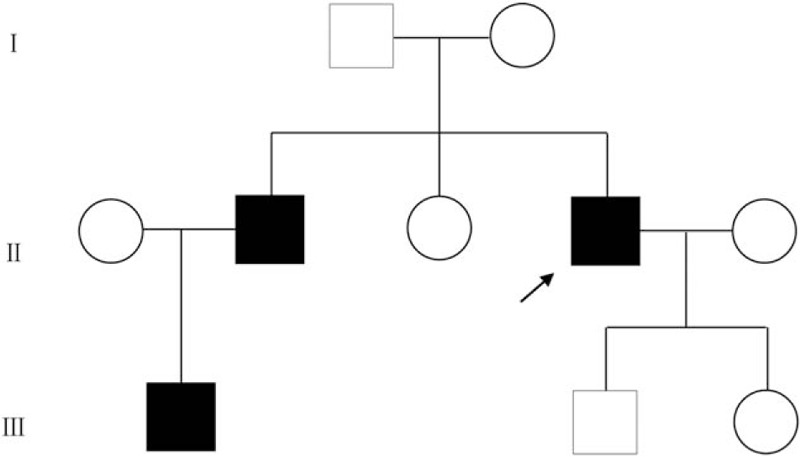
Pedigree of the family with Carney complex. Arrow indicates the proband.

A family pedigree across 3 generations was constructed based on personal communication with the patient (Fig. 3). The brother of the proband and the brother's son (the nephew of the proband) were both confirmed with the diagnosis of atrial myxoma and underwent surgical resection. The nephew underwent surgical resections twice, just like the proband. The father of the proband encountered sudden death at the age of 43 without any diagnosed disease.

### 2.5 Whole exome sequencing

**Figure 4:**
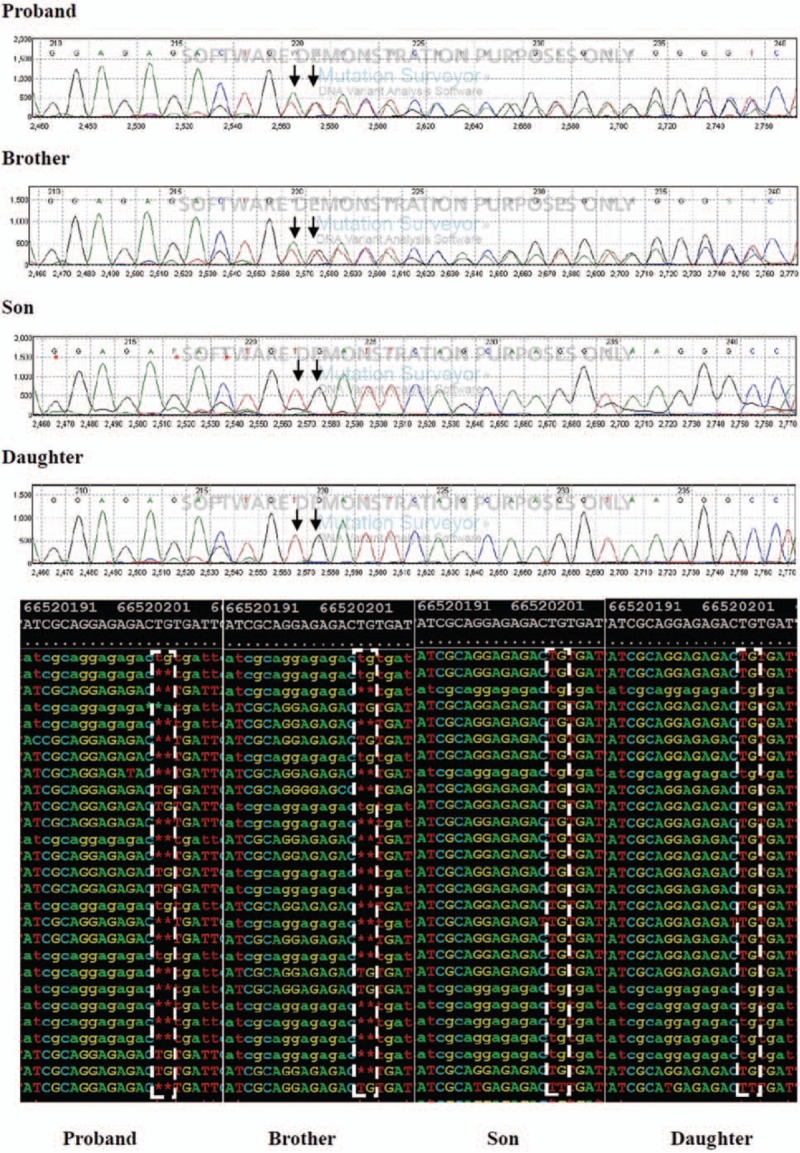
Whole exome sequencing results of the patients and his family. Both CNC cases in the family (the proband and the brother) carried the same PRKAR1A exon 5 mutation (c.491-492delTG), and the healthy son and daughter (of the proband) did not carry the mutation. CNC = Carney complex, PRKAR1A = protein kinase type I-alpha regulatory subunit.

In order to find the mutation and confirm the diagnosis, whole exome sequencing was performed for the proband, his brother, and his 2 children by GENEWIZ corporation (Suzhou, China) to make the diagnosis. Due to the inaccessibility of the blood sample, the nephew's genetic test could not be completed. An analysis of the patient's genomic DNA obtained from peripheral blood identified a p.E17X (c.491-492delTG) mutation in PRKAR1A, which encodes the type Iα regulatory subunit of protein kinase A (Fig. 4). The same point mutation in PRKAR1A was also found in the brother, but not in the children of the proband, who had no atrial myxoma. According to the diagnostic criteria of CNC (Table 1),^[4]^ since 3 major criteria and one supplemental criterion were met, the CNC diagnosis of the family was effectively established.

**Table 1:**
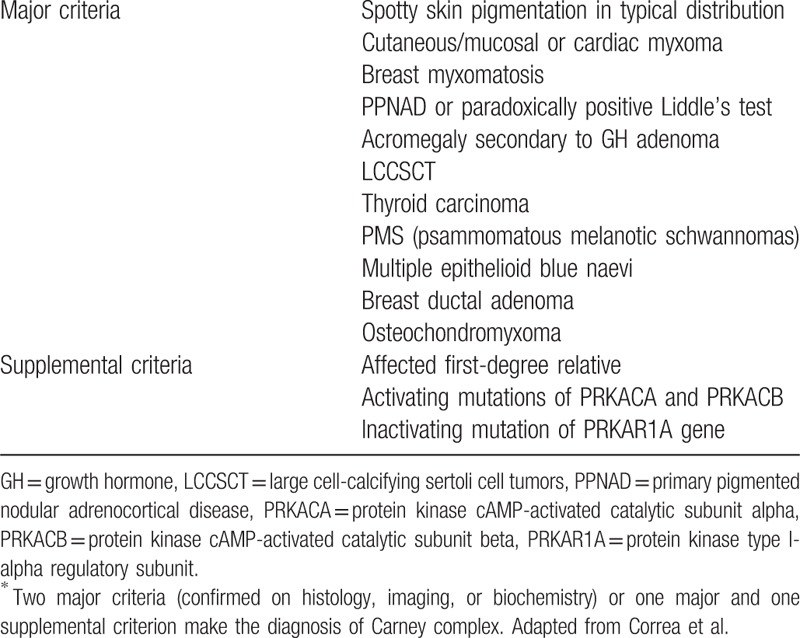
Carney complex: diagnostic criteria^∗^.

## 3 Discussion

CNC is a rare autosomal dominant disease, first reported by Carney, in 1985. It is characterized by cardiac and cutaneous myxoma, spotty skin pigmentation, and endocrine over-reactivity. The syndrome was found to be responsible for many endocrine tumors in addition to myxoma. To confirm the diagnosis of CNC, patients must meet 2 major criteria or 1 major and 1 supplemental criterion (Table 1).^[4]^ Thus, a patient with a histologically proven cardiac myxoma can be diagnosed with CNC when he/she meets an additional major criterion, including a recognized gene activation/inactivation or existence of an affected first-degree relative. In this Chinese CNC-affected family, the proband had cardiac myxoma, skin pigmentation on his upper lip as well as right hand, and multiple hypoechoic thyroid nodules. Similarly, the nephew and uncle exhibited cardiac myxoma and skin pigmentation.

The mutation in the cAMP-dependent protein kinase A (PKA) regulatory (R) subunit 1 (PRKAR1A) gene on loci 24.1–24.3 of the long arm of chromosome 17 has been identified as a cause of CNC.^[5]^ More than 1000 CNC patients have been reported, of whom approximately 70% are familial cases. The PRKAR1A mutation is found in more than 70% of CNC patients, and >100 different mutations have been reported throughout the coding region of PRKAR1A.^[6]^ Horvath reviewed all the known PRKAR1A mutations and has established an online database of the same (http://PRKAR1A.nichd.nih.gov).^[7]^ However, little is known about the role of this mutation in the development of CNC in Asian populations. Using target exome capture sequencing, we identified the mutation in this CNC kindred in PRKAR1A exon 5 that included a deletion of TG at chr17 66520201-66520211.

PRKAR1A encodes the type 1A R subunit of PKA or cAMP-dependent protein kinase. The inactive PKA enzyme consists of a tetramer of 2 homo- or heterodimers of R subunits (PRKAR1A, PRKAR1B, PRKAR2A, and PRKAR2B) and a homodimer of 2 catalytic subunits (from a choice of 4 molecules: PRKACA, PRKACB, PRKACG, and PRKX). Activation of PKA occurs upon the binding of 2 molecules of cAMP to each R subunit,^[8]^ followed by the dissociation of the holoenzyme and the release of the active catalytic subunit. This in turn phosphorylates a series of downstream cellular target genes.^[9]^ PRKAR1A haploinsufficiency leads to an increase in total (and not only PKA-specific) cAMP-stimulated kinase activity.^[5]^

Previous studies have explained the increment of cAMP signaling with 2 major pathways. One mechanism is that an increased intracellular C to R subunit ratio and a higher amount of free catalytic subunits which phosphorylate downstream targets were caused by R-Iα haploinsufficiency. The other mechanism is that R-Iα haploinsufficiency can result in an upregulation of subunits of PKA tetramer, such as type I (PRKAR1B) and type II (PRKAR2A or PRKAR2B).^[10,11]^

Patients with cardiac myxoma were associated with CNC experience recurrence in up to 30% of cases, whereas in the total population of cardiac myxoma, reported rates of recurrence vary between 3% and 6%.^[3]^ Cases of up to 4 and 7 recurrences in single patients have been reported.^[4]^ Two out of the 3 patients in our series had tumor recurrence, and in both cases at the same location as the first tumor. Thorough and complete physical examinations, especially the skin evaluation and thyroid examination, are compulsory in the diagnosis and interval follow-up for CNC patients.^[12]^ Considering the highly risk of recurrence of cardiac myxoma, every patient diagnosed with CNC shall be given clinical surveillance by biannual TTE examination.^[13]^ For male CNC patients, large cell-calcifying Sertoli cell tumors (LCCSCT) detection by testicular ultrasound is strongly recommended because approximately 75% proportion of male CNC patients are accompanied by this disease. Laboratory test such as serum growth hormone (GH), plasma prolactin (PRL), insulin-like growth factor 1 (IGF1), and urinary free cortisol are often tested as the screening biochemical indicators.^[14]^

## 4 Conclusions

A familial case of 3 first-degree relatives with cardiac myxoma was reported, who showed the classic symptoms of CNC including cardiac myxoma, skin pigmentation, and multiple hypoechoic thyroid nodules, which was confirmed by whole exome sequencing. Recurrence of cardiac myxoma was found in 2 patients of this family, with one patient had late recurrence at 5 years after the operation, another patient presented with his third recurrence. For patients with familial cardiac myxoma, the diagnosis of CNC should be considered, which can be established when the patient is accompanied by other major criteria. Once the patient is diagnosed with CNC, periodical follow-up, especially the surveillance by echocardiography is strongly recommended for himself, as well as his first-degree relatives, in case of recurrence.

## 5 Author contributions

**Conceptualization:** G. Ji, L. Wang.

**Data curation:** L. Wang, Q. Xue, Qing Wang.

**Formal analysis:** X. Sun.

**Funding acquisition:** Q. Xue.

**Investigation:** Q. Xue.

**Methodology:** X. Sun.

**Resources:** Y. Zhou.

**Software:** X. Sun.

**Supervision:** G. Ji, Z. Wang.

**Validation:** Q. Xue.

**Visualization:** Y. Zhou.

**Writing – original draft:** L. Wang.

**Writing – review & editing:** Qing Wang.

## References

[1] Stratakis CA Kirschner LS Carney JA Genetics of endocrine disease—clinical and molecular features of the Carney complex: diagnostic criteria and recommendations for patient evaluation. J Clin Endocrinol Metab 2001;86:4041–6. 11549623 10.1210/jcem.86.9.7903

[2] Shetty Roy A Radin M Sarabi D Familial recurrent atrial myxoma: Carney's complex. Clin Cardiol 2011;34:83–6. 21298650 10.1002/clc.20845 PMC6652706

[3] Shah I Dearani J Daly R Cardiac myxomas: a 50-year experience with resection and analysis of risk factors for recurrence. Ann Thorac Surg 2015;100:495–500. 26070596 10.1016/j.athoracsur.2015.03.007

[4] Correa R Salpea P Stratakis C Carney complex: an update. Eur J Endocrinol 2015;173:M85–97. 26130139 10.1530/EJE-15-0209 PMC4553126

[5] Kirschner L Carney J Pack S Mutations of the gene encoding the protein kinase A type I-alpha regulatory subunit in patients with the Carney complex. Nat Genet 2000;26:89–92. 10973256 10.1038/79238

[6] Bertherat J Horvath A Groussin L Mutations in regulatory subunit type 1A of cyclic adenosine 5′-monophosphate-dependent protein kinase (PRKAR1A): phenotype analysis in 353 patients and 80 different genotypes. J Clin Endocrinol Metab 2009;94:2085–91. 19293268 10.1210/jc.2008-2333 PMC2690418

[7] Horvath A Bertherat J Groussin L Mutations and polymorphisms in the gene encoding regulatory subunit type 1-alpha of protein kinase A (PRKAR1A): an update. Hum Mutat 2010;31:369–79. 20358582 10.1002/humu.21178 PMC2936101

[8] Bruystens JGH Wu J Fortezzo A PKA Rl alpha homodimer structure reveals an intermolecular interface with implications for cooperative cAMP binding and Carney complex disease. Structure 2014;22:59–69. 24316401 10.1016/j.str.2013.10.012 PMC3963464

[9] Bossis I Stratakis CA Minireview: PRKAR1A: normal and abnormal functions. Endocrinology 2004;145:5452–8. 15331577 10.1210/en.2004-0900

[10] Rothenbuhler A Stratakis CA Clinical and molecular genetics of Carney complex. Best practice & research. Clin Endocrinol Metab 2010;24:389–99. 10.1016/j.beem.2010.03.003 20833331

[11] Stergiopoulos SG Stratakis CA Human tumors associated with Carney complex and germline PRKAR1A mutations: a protein kinase A disease!. Febs Lett 2003;546:59–64. 12829237 10.1016/s0014-5793(03)00452-6

[12] Siordia JA Medical and surgical management of Carney complex. J Card Surg 2015;30:560–7. 25996461 10.1111/jocs.12575

[13] Jain S Maleszewski JJ Stephenson CR Current diagnosis and management of cardiac myxomas. Expert Rev Cardiovasc Ther 2015;13:369–75. 25797902 10.1586/14779072.2015.1024108

[14] Schmidt C Doi A Ura M Familial atrial myxoma: three related cases at an Australian tertiary institution. Ann Thorac Cardiovasc Surg 2017;23:203–6. 28367853 10.5761/atcs.cr.16-00169 PMC5569256

